# Arrhythmic Risk Stratification in Non-Ischaemic Cardiomyopathy: The Role of Cardiac Magnetic Resonance Imaging

**DOI:** 10.1007/s11886-026-02435-8

**Published:** 2026-10-23

**Authors:** Ryan I. Sia, Daniel J. Hammersley

**Affiliations:** 1 Faculty of Life Sciences and Medicine, King’s College London, London, UK; 2 British Heart Foundation Centre of Research Excellence, School of Cardiovascular and Metabolic Medicine and Sciences, King’s College London, London, UK; 3 Department of Cardiology, King’s College Hospital NHS Foundation Trust, Denmark Hill, London, SE5 3RS UK

**Keywords:** Cardiac magnetic resonance imaging, Risk stratification, Arrhythmias, Non-ischaemic cardiomyopathy

## Abstract

**Purpose of Review:**

This review aims to evaluate the contemporary and evolving role of cardiac magnetic resonance (CMR) imaging in arrhythmic risk stratification for patients with non-ischaemic cardiomyopathy, specifically focussing on dilated cardiomyopathy (DCM) and non-dilated left ventricular cardiomyopathy (NDLVC). This review synthesises current data on late gadolinium enhancement (LGE), parametric mapping, strain imaging and artificial intelligence (AI), and contextualises these among emerging genetic risk stratification approaches.

**Recent Findings:**

Left ventricular ejection fraction has been the cornerstone for guideline-based arrhythmic risk stratification in patients with DCM, despite limited precision. Observational data and meta-analyses have established the presence of replacement fibrosis through LGE-CMR as a powerful independent predictor of ventricular arrhythmias (VAs) and sudden cardiac death. Crucially, the absence of LGE identifies a low-risk cohort. Much recent focus has been on understanding whether more precise characterisation of scar can further enhance arrhythmic risk stratification, for example through scar quantification. Although data exists supporting a potential role for T1 mapping for arrhythmic risk prediction in this cohort, further research is required to support its widespread adoption. Emerging multimodal approaches using artificial intelligence and personalised computational “digital twins” are areas of important interest, offering scope for integration of imaging, electrocardiographic and genomic data layers.

**Summary:**

Arrhythmic risk stratification in non-ischaemic cardiomyopathies remains challenging despite significant advances. Progressive refinement in precision has accompanied a shift from a restrictive, functional assessment towards an integrated, substrate-driven precision model. CMR represents the cornerstone of this evolution.

## Introduction

The term ‘non-ischaemic cardiomyopathies’ (NICM) describes a group of conditions arising from intrinsic disease of the myocardium. Common to this highly heterogeneous group of patients is an underlying dynamic risk of sudden cardiac death (SCD), which most commonly occurs in this population due to ventricular arrhythmias (VAs). Estimating the risk of SCD, and implementing therapies to mitigate this, represents a critical component of the clinical management of these patients. These conditions are routinely classified based on their phenotype. Revised nomenclature from the 2023 European Society of Cardiology (ESC) Guidelines for the management of cardiomyopathies defines five distinct cardiomyopathy phenotypes. The focus of this review is the arrhythmic risk stratification of patients with dilated cardiomyopathy (DCM) and non-dilated left ventricular cardiomyopathy (NDLVC), among whom there exists substantial clinical overlap.

DCM is among the most prevalent of NICMs, affecting approximately 1 in 250 individuals, and is defined as a heterogeneous primary myocardial disease characterised by left ventricular (LV) or biventricular dilation and systolic dysfunction, not explained solely by abnormal loading conditions or coronary artery disease [1]. NDLVC, a distinct clinical entity to DCM, is defined by the presence of non-ischaemic LV scarring or fatty replacement, with or without global LV hypokinesis, but in the absence of LV dilation [2]. Previous nomenclature utilised the term “hypokinetic non-dilated LV cardiomyopathy”, which described the cardiomyopathy subgroup with contractile impairment without cavity dilation [3]. The 2023 ESC Guidelines replaced this term with NDLVC, expanding the definition to encompass patients with or without systolic dysfunction, and formally recognising non-ischaemic myocardial scarring or fatty replacement as the critical element of this cardiomyopathy subgroup.

While epidemiological studies have shown a temporal linear decline in the rate of SCD in non-ischaemic DCM patients [4], the risk of arrhythmias and SCD in NICM remains significant. Among patients with NICM and a left ventricular ejection fraction (LVEF) < 35%, approximately 30% of deaths are arrhythmic in aetiology [5].

Extensive evidence exists to support the pathobiological mechanisms, notably scar-related macro-reentry, underlying VAs in DCM and NDLVC [6]. Pathological processes like fibro-fatty replacement and diffuse interstitial collagen deposition disrupt the uniform propagation of electrical conduction through the systemic uncoupling of adjacent cardiomyocytes [7, 8]. These disruptions increase tissue resistance and create zones of slow conduction and functional block across the myocardium [9]. Within this fibrotic architecture, surviving myocyte bundles interspersed between dense scar form discrete arrhythmia channels – corridors of preserved but functionally abnormal conduction that serve as the electrophysiological substrate for stable reentrant circuits [10, 11]. The structural heterogeneity imposed by progressive fibrosis creates the conditions necessary for both the initiation and perpetuation of VAs, including ventricular fibrillation (VF). Although there has been a correlation between scar burden and LVEF, it is feasible to have significant scar and arrhythmic substrate in patients with mildly reduced LVEF. Consequently, characterising this arrhythmogenic substrate is of paramount importance for accurate risk stratification.

For decades, the cornerstone of arrhythmic risk stratification and candidacy for primary prevention implantable cardioverter-defibrillator (ICD) implantation has been centred on the LVEF, where a threshold of ≤ 35% has been universally adopted, alongside New York Heart Association (NYHA) class [12]. The foundational evidence supporting LVEF as the primary criteria for ICD candidacy stems from landmark trials such as DEFINITE and SCD-HeFT, alongside its adoption among the inclusion criteria in randomised controlled ICD trials (Table 1). While these trials demonstrated a mortality benefit for primary prevention ICDs in mixed or non-ischaemic cohorts, they were conducted in an era preceding the application of modern, highly effective heart failure pharmacotherapy.

**Table 1:** Trials that determined the role of ejection fraction (EF) in implantable cardioverter-defibrillator (ICD) candidacy

Study		*N*			Inclusion Criteria		Intervention	Median Follow-Up		All-Cause Mortality		SCD
CAT (2002) [13]		104			LVEF < 30% NYHA 2–3		ICD vs. OMT	23 months		Terminated early		
AMIOVIRT (2003) [14]		103			LVEF ≤ 35% NYHA 1–3 NSVT		ICD vs. amio	24 months		Terminated early		
DEFINITE (2004) [15]		458			LVEF < 36% NYHA 1–3 NSVT or PVCs		ICD vs. OMT	29 months		I: 12.2%, C: 17.4% HR 0.65; 95% CI 0.40–1.06 *p* = 0.08		I: 1.3%, C:6.1% HR 0.20; 95% CI 0.06–0.71 *P* = 0.006
SCD-HeFT (DCM cohort) (2005) [16]		1211			LVEF < 35% NYHA 2–3		ICD vs. OMT vs. amio	46 months		I: 21.4%, C: 27.9% (5 yrs) HR 0.73; 95% CI 0.50–1.07 *p* = 0.06		
DANISH (2016) [17]		1116			LVEF < 35% NYHA 2–3 (4 if CRT) NT-pro-BNP>200pg/ml		ICD vs. OMT	68 months		I: 21.6%, C: 23.4% HR 0.87; 95% CI 0.68–1.12 *p* = 0.28		I: 4.3%, C: 8.2% HR 0.50; 95% CI 0.31–0.82 *p* = 0.005

Reproduced with permission from: Halliday et al. Circulation [Internet]. 2017;136:215–31. Available from: http://circ.ahajournals.org/lookup/doi/10.1161/CIRCULATIONAHA.116.0271340 [18]

Key: *Amio* Amiodarone, *C* Optimal medical therapy arm, *CI* Confidence interval, *CRT *Cardiac resynchronisation therapy, *HR *Hazard ratio, *I* Implantable cardioverter defibrillator therapy arm, *ICD* Implantable cardioverter defibrillator, *LVEF *Left ventricular ejection fraction, *NYHA *New York Heart Association, *NT-pro-BNP *N-terminal-pro-peptide brain natriuretic peptide, *NSVT *Non-sustained ventricular tachycardia, *PVCs *Premature ventricular complexes, *OMT *Optimal medical therapy, *SCD *Sudden cardiac death

However, more recent clinical trials have brought into question the sensitivity of LVEF-based selection in identifying high-risk individuals. Notably, findings from major trials such as DANISH, which demonstrated ICD implantation did not improve survival in NICM patients with an LVEF ≤ 35%, have highlighted this [16]. This has been further supported by more recent studies, including a large 2024 observational study where two key findings were made: (1) an LVEF < 35% failed to reach significance as a predictor of SCD or VAs when myocardial fibrosis was included as a co-variable, suggesting LVEF may merely act as a surrogate for underlying myocardial scarring, and (2) the absence of myocardial fibrosis on visual assessment virtually excluded the risk of SCD and ventricular arrhythmias in the long term, regardless of the patient’s LVEF (negative predictive value: 98%) [19].

**Fig. 1:**
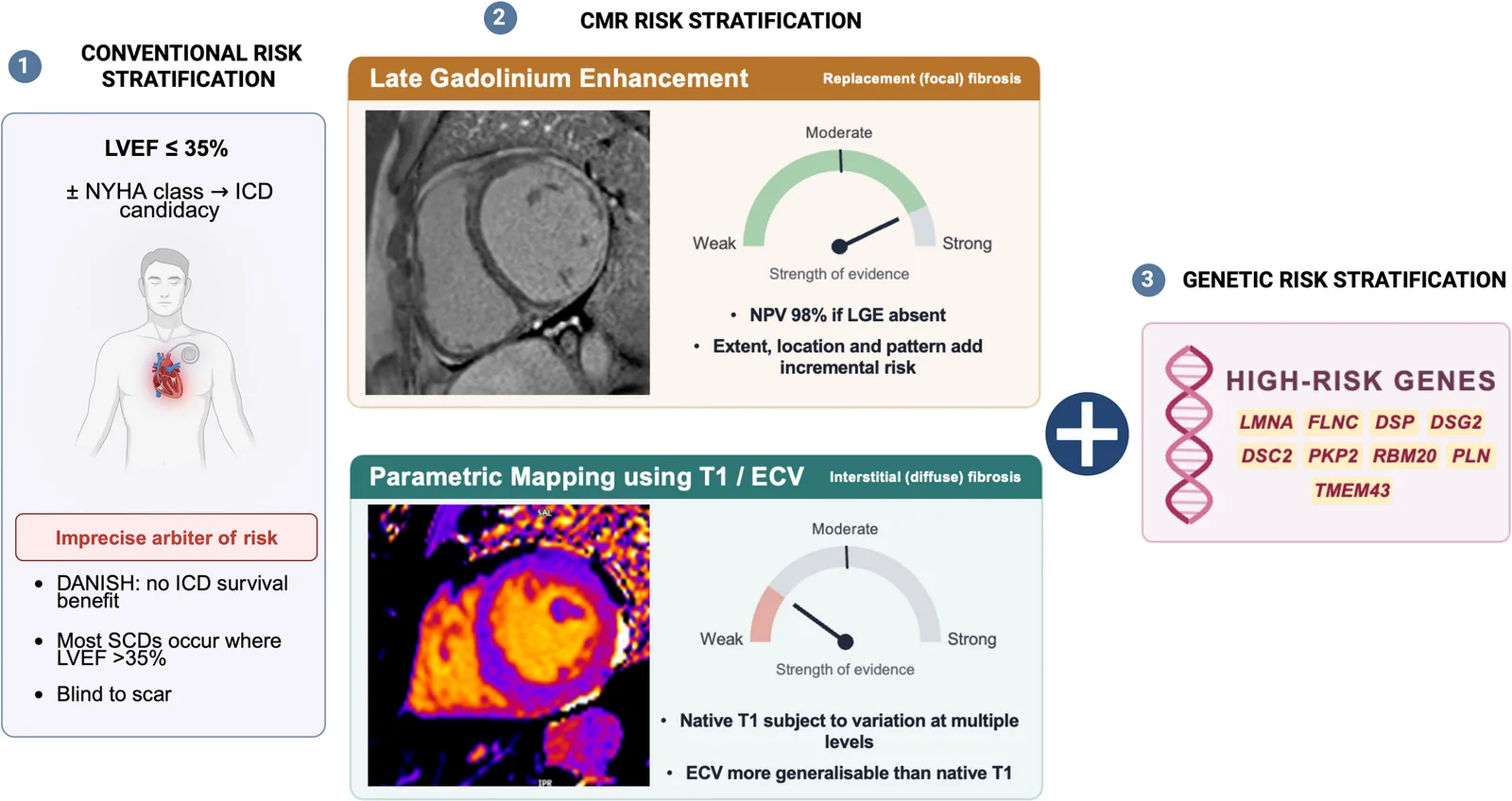
From LVEF-based to substrate-based arrhythmic risk stratification in non-ischaemic cardiomyopathy. *Created in BioRender. Sia*,* R. I. (2026). *https://BioRender.com/in7w66m. Key: CMR – cardiac magnetic resonance, *DSC2* – desmocollin 2, *DSG2* – desmoglein 2, *DSP* – desmoplakin, ECV – extracellular volume, *FLNC* – filamin C, ICD – implantable cardioverter defibrillator, LGE – late gadolinium enhancement, *LMNA* – lamin A/C, LVEF – left ventricular ejection fraction, NICM – non-ischaemic cardiomyopathy, NPV – negative predictive value, NYHA – New York Heart Association, *PKP2* – plakophilin 2, *PLN* – phospholamban, *RBM20* – RNA-binding motif protein 20, SCD – sudden cardiac death, *TMEM43* – transmembrane protein 43

The legacy of the DANISH trial and subsequent studies lies in the fact that LVEF is a profoundly imprecise tool for arrhythmic risk stratification, where LVEF has never been shown to be a reliable predictor of VAs in NICM [20]. While a low LVEF is a robust marker of advanced myocardial disease and global mortality risk, it does not effectively discriminate patients whose main threat is an arrhythmic event from those who are more likely to succumb to pump failure. Indeed, most SCD events in the general population occur in individuals with an LVEF > 35% (who would not have fulfilled ICD implantation criteria), while conversely, many patients with severely depressed LVEF never experience malignant arrhythmias [21–23]. Collectively, this highlights an urgent need for more precise stratification tools (Fig. 1).

Cardiac magnetic resonance imaging (CMR) has emerged as the gold standard non-invasive modality for comprehensive myocardial tissue characterisation [24, 25]. Through methods such as late gadolinium enhancement (LGE), parametric mapping and advanced functional deformation tracking, CMR provides a high-fidelity spatial and temporal representation of the myocardium. Recent ESC and ACC/AHA guidelines have also explicitly recognised the importance of CMR-derived metrics in refining risk stratification algorithms for NICM [26]. This review synthesises the contemporary evidence surrounding CMR modalities in evaluating arrhythmic risk in DCM and NDLVC, exploring the clinical utility of CMR parameters, the integration of artificial intelligence (AI) and positions these alongside the emerging role of clinical genetics.

## Cardiovascular MRI Parameters and Clinical Utility

### Late Gadolinium Enhancement

Gadolinium-based contrast agents are extracellular paramagnetic agents which distribute freely into extracellular space but are unable to cross intact myocyte cell membranes. In areas of prior myocardial necrosis, the cellular architecture is replaced by dense, biochemically inert collagen networks, resulting in an expanded extracellular space and thus, delayed contrast washout [27]. Electrophysiologically, replacement fibrosis serves as the requisite anatomical substrate for abnormal electrical conduction and arrhythmias [7, 28]. These regions of replacement fibrosis appear hyperintense on specialised T1-weighted inversion recovery sequences, against the dark background of healthy myocardium [29].

The prognostic utility of LGE in NICM is supported by a large volume of observational data. Gulati et al. initially demonstrated a powerful association between the presence of mid-wall LGE and SCD in NICM, with this remaining an independent predictor after adjustment for LVEF and conventional clinical variables [30]. This has been further replicated and validated in other cohorts. Most recently, an exhaustive systematic review and meta-analysis by Eichhorn et al. aggregated data from 103 studies encompassing nearly 30,000 patients with DCM. The meta-analyses established the binary presence of LGE – seen in approximately 40% of patients with NICM – was a strong predictor of adverse outcomes, displaying its strongest association with VAs and SCD (HR 2.69) among all endpoints evaluated [5, 31]. Other meta-analyses have corroborated this, where the mere presence of LGE in DCM increases mortality three-fold and the risk of SCD or aborted SCD four-fold, compared to matched patients without LGE [32, 33] (Fig. 1).

Beyond visual presence, there has been much interest in whether a deeper phenotypic characterisation of scar provides superior risk stratification. One such characteristic is the fibrosis mass/extent, where a number of studies have demonstrated that higher scar mass/extent is associated with greater arrhythmic risk [19, 34]. A limitation to this approach relates to a lack of uniformity in scar quantification methods and intrinsic differences that may emerge between scar quantification undertaken on patients scanned using different magnetic field strengths, using different contrast agents or between those analysed across different software platforms. One study demonstrated that a small amount of LGE (e.g. 0% to 0.71% of LV mass) was associated with a substantial increase in risk compared to patients without LGE, suggesting a non-linear dose-response relationship [21, 35, 36]. A more recent study proposed a dichotomised cutoff, identifying an LGE burden of ≥ 7.5% as the strongest predictor of VAs [37]. A larger 2-centre study of 1,714 patients with NICM quantified total fibrosis mass, where it was found patients with > 10 g of total fibrosis had a nine-fold increased hazard for a composite endpoint of SCD, aborted SCD or VF (HR 9.17; 95% CI 4.64-18.1) compared to patients with no fibrosis [19].

In addition to overall burden, fibrosis position may play an important role in risk stratification. In DCM, septal involvement is most commonly observed, where LGE predominantly manifests in a mid-wall distribution [36, 38, 39]. Indeed, mid-wall LGE, particularly localised to the basal septal and lateral walls, has been shown to strongly increase the risk of life-threatening VAs and SCD risk and mortality [30, 35, 40]. This has been reflected in a large contemporary European dataset: DERIVATE registry’s updated Risk Score 2.0, affirming that the presence and location of mid-wall scar confer additive, independent risk [41].

Beyond mass and position, the scar pattern might hold additional prognostic significance. Advanced imaging analyses have identified a particularly malignant topological variant known as the “ring-like” LGE pattern, observed in both DCM and NDLVC patients [39]. This is characterised by a contiguous, circumferential mid-wall or subepicardial scarring spanning multiple myocardial segments [42, 43]. This pattern has been associated with a high burden of VAs and increased risk of malignant arrhythmias, but importantly, not an impairment in LVEF, further highlighting the limitations of LVEF as a proxy for ICD implantation [39].

Nevertheless, there remains a lack of research into the diagnostic and prognostic value of LGE in patients who fall outside the traditional bounds of severe systolic dysfunction. This includes patients with “mild” DCM (characterised by an LVEF > 40%) and those with “recovered/improved” DCM (also termed HFimpEF: heart failure with improved ejection fraction), whose LVEF has improved following treatment with guideline-directed medical therapy (GDMT) [44]. Notably, Halliday et al. demonstrated that the presence of LGE is strongly associated with an increased risk of SCD in DCM patients with mild and moderate left ventricular systolic dysfunction [35]. Crucially, LVEF is an inherently dynamic parameter that often changes over time in these conditions. As a result of this, a single snapshot of functional remodelling cannot be equated with a reduced risk of SCD [45–47].

### Parametric Mapping

While the clinical and prognostic validity of LGE for mapping replacement fibrosis is firmly established, this correlates histologically with replacement fibrosis and does not capture interstitial fibrosis. While replacement scar signifies dense, irreversible collagen deposition, interstitial fibrosis represents an earlier, globally distributed remodelling phase that remains potentially reversible. CMR parametric mapping, specifically T1 mapping and the derivation of the extracellular volume (ECV) fraction, permits quantitative assessment of myocardial interstitial fibrosis. Native T1 mapping evaluates the relaxation time of myocardial tissue; prolonged native T1 relaxation times, often coupled with shortened post-contrast T1 times, are indicative of expanded interstitial volume, serving as a surrogate for fibrotic remodelling [48]. By integrating both pre- and post-contrast measurements, clinicians can derive the ECV fraction, which acts as a sensitive proxy for the percentage of the interstitial space expanded by fibrosis.

The prognostic relevance of CMR parametric mapping in NICM was evaluated by Puntmann et al. in a multicentre study of 637 patients. This demonstrated that native T1 and ECV fraction were associated with mortality and heart failure outcomes. In multivariable analysis, native T1 emerged as the sole independent predictor of the study’s endpoints [49]. It is important to recognise, however, that these endpoints were defined as all-cause mortality and a heart failure composite, rather than arrhythmic events. Subsequent research has bridged this gap by studying parametric mapping parameters in relation to VAs. Chen et al. demonstrated that native T1 is an independent predictor of appropriate ICD therapy or documented sustained VAs in patients with NICM, where patients faced a 1.4-fold increased risk of the primary arrhythmic endpoint for every 10 ms increment in non-contrast native T1 [50].

This was further corroborated by Li et al. in a large cohort of 659 patients with DCM, which found native T1 and ECV possess significant prognostic value, and importantly, established a combined assessment of LGE, T1 and ECV provides optimal risk stratification [51]. This study also noted that parametric mapping provides orthogonal risk discrimination for patients who do not exhibit regional scarring on LGE. In LGE negative patients, mean native T1 and mean ECV fraction were the only independent variables associated with cardiovascular death. It is important to note that this study was not focused on arrhythmic endpoints, and so it remains unknown whether ECV may have utility in arrhythmic risk stratification of LGE negative NICM patients. Studies specifically probing this would be challenging to conduct due to the low incidence of VAs in this subgroup.

In a further study, Di Marco et al. found that an ECV threshold of ≥ 30% was significantly associated with an elevated risk of VAs in patients with NICM, demonstrating a 14.1-fold increased risk even after adjustment for the presence of LGE and an LVEF ≤ 35% [52]. Their analysis of 618 patients with NICM showed incorporating this ECV threshold into risk models reclassified approximately 32% of the study population compared to using LVEF alone. Notably, this approach identified that 71% of patients who would traditionally have been classified as high-risk based on an LVEF ≤ 35% were actually at low risk for arrhythmic events. A simple clinical model integrating LGE, LVEF ≤ 35%, and ECV ≥ 30% achieved a high predictive performance (Harrell’s C-statistic: 0.820 to 0.885). Interestingly, native T1 mapping and global longitudinal strain (GLS) in this specific cohort did not provide additional independent prognostic value for the arrhythmic endpoint [52].

A 2025 meta-analysis by Marchini et al. evaluating T1 mapping and ECV in non-ischaemic DCM patients demonstrated both parameters are independently associated with major adverse cardiovascular events (MACE) and arrhythmic outcomes [53]. Each 1% absolute increase in ECV was associated with a 37% increase in the risk for MACE (HR 1.37; 95% CI 1.29–1.44), while each 10 ms elevation in native T1 time was associated with a 7% increase in the risk of MACE (HR 1.07; 95% CI 1.04–1.09). Native T1 mapping also demonstrated significant prognostic value for arrhythmic-related events specifically, with a pooled HR of 1.09 (95% CI 1.07–1.12) per 10 ms increase.

An important caveat in interpreting native T1 data is its limited external generalisability. Native T1 values are strongly dependent on magnetic field strength, being substantially longer at 3T than at 1.5T, and are further influenced by the mapping sequence (e.g. MOLLI, ShMOLLI or SASHA), vendor and acquisition parameters [54]. Consequently, normal reference ranges must be established for each scanner and sequence and validated in healthy controls at each centre. Absolute native T1 thresholds derived in one cohort therefore cannot readily be transferred to another, and a single universally applicable cutoff is unlikely to be achievable. By contrast, ECV is derived from the ratio of pre- and post-contrast changes in myocardial and blood-pool T1, corrected for haematocrit, which partially normalises for field strength and sequence-related differences [54]. This makes ECV less subject to variation in magnetic field strength and parametric mapping sequence (Fig. 1).

Despite the evidence surrounding T1 and ECV, there is currently a paucity in the literature evaluating T2 mapping of arrhythmic risk stratification in DCM and NDLVC. While T2-weighted imaging is essential for identifying myocardial oedema in acute inflammatory states like myocarditis, its role in characterising the chronic pro-arrhythmic substrate of DCM remains unclear.

### Left Ventricular Strain Imaging

Myocardial deformation analysis, widely known as strain imaging, evaluates myocardial shortening, thickening and lengthening throughout the cardiac cycle. Feature-tracking cardiovascular magnetic resonance (CMR-FT) has emerged as a highly reproducible method for this, by utilising standard steady-state free precession (SSFP) cine images to mathematically track tissue voxel displacement over time, thereby calculating the precise regional deformation of myocardial fibres by quantifying myocardial shortening, thickening and lengthening [55, 56].

Global longitudinal strain (GLS) evaluates the shortening of subendocardial, longitudinally-oriented myocardial fibres [57]. These fibres are particularly susceptible to early microvascular ischaemia, elevated wall stress and initial fibrotic infiltration. In one large multicentre study involving 1,012 patients with DCM by Romano et al., impaired CMR-derived LV GLS was proven to be a powerful, independent predictor of mortality, incremental to LVEF and LGE. After adjustment for clinical parameters, LVEF and the extent of LGE, every 1% absolute reduction in GLS conferred a nearly 89% increased relative risk of all-cause mortality (HR 1.89 per %; *p* < 0.001) [58]. However, it is important to note that no data on arrhythmic events were reported in this study. A further study involving 355 DCM patients with mildly reduced EF (LVEF ≥ 40%) found LV GLS was not associated with SCD, and LGE presence was the only predictor of SCD [59]. Currently there is a lack of data to suggest that LV GLS may be a useful imaging parameter in arrhythmic risk stratification in NICM. This follows mechanistically, as LV GLS is ultimately a metric of contractile performance, and hence is not a direct measure of arrhythmic substrate.

### Artificial Intelligence

While conventional CMR analysis has substantially advanced the field, it remains constrained by factors such as inter-observer variability, time-intensive post-processing demands and the inherent biological limitation of human visual assessment of LGE. The burgeoning fields of AI, deep learning (DL) and machine learning (ML) are shifting CMR to an automated, high-throughput and high-dimensional investigation.

One such advance made in arrhythmic risk stratification is the transition from human-derived algorithms to deep multimodal learning architectures. SCD is inherently a multifactorial event, influenced by complex interactions between genetics, structural pathology, electrical conduction, systemic comorbidities, and patient circumstance. Recognising this complexity, Lai et al. have recently developed a Multimodal AI for Ventricular Arrhythmia Risk Stratification (MAARS) model. It is important to note that while MAARS has only been developed and validated on hypertrophic cardiomyopathy (HCM) patients, this serves as a proof-of-concept for the development of similar models in DCM and NDLVC patients. MAARS is an AI framework utilising an advanced transformer-based neural network in order to integrate contrast-enhanced CMR images with electronic health records, clinical reports and continuous ECG parameters. This approach allows for the discovery of previously hidden, highly complex architectural fibrosis patterns that precipitate arrhythmias, where the model is capable of identifying subtle, high-risk ECG features. Indeed, MAARS has demonstrated strong predictive accuracy for SCD due to arrhythmias, yielding area under the receiver operating characteristic curve (AUROC) values approaching 0.89 and vastly outperforming existing 2024 ACC/AHA and 2022 ESC guidelines [60].

Another growing application is the development of the “digital twin”, a highly personalised, in silico mathematical model of a patient’s heart [61, 62]. These dynamic virtual patient representations integrate multi-modal data to simulate clinical scenarios and treatment strategies, allowing for the forecasting of clinical outcomes. One such application was demonstrated by O’Hara et al., who developed personalised virtual-heart replicas to assess SCD. By fusing data from two different CMR modalities – LGE to identify focal scar and T1 mapping to visualise diffuse fibrosis – high-fidelity digital models of the patient-specific arrhythmogenic substrate were created. These digital twins achieved 80.1% accuracy in forecasting future VA events, significantly outperforming conventional clinical risk predictors from existing guidelines such as the ESC [63].

Beyond these static mechanistic models, the future of arrhythmic risk stratification lies in the transition to “active” digital twins that maintain a continuous cyber-physical connection with the patient. This next generation of technology will integrate real-time data from wearables such as smartwatches, alongside historical data like genomics and medical records. The integration of these multimodal data streams into deep learning architectures and generative AI models may allow the synthesis of missing cardiac data and the simulation of 3D scarring patterns [64]. By doing so, clinicians can move towards a dynamic, individualised risk profile that evolves in real-time as a patient’s condition changes or new therapies are introduced.

## Role of Genetics

Given that it is now recognised that between 20% and 30% of DCM cases are familial in origin, of which 40% have an identifiable monogenic cause, identifying genetically-predisposed individuals at a higher risk of malignant VAs is paramount for arrhythmic risk stratification [65]. Extensive research has isolated a specific subset of genes associated with a malignant clinical trajectory. These high-risk loci encompass variants affecting the nuclear envelope (e.g. *LMNA*, *TMEM43*), cytoskeletal and Z-disk proteins (e.g. *FLNC*), desmosomal complexes (e.g. *DSP*, *DSG2*, *DSC2*, *PKP2*), RNA-splicing elements (e.g. *RBM20*) and calcium-handling proteins (e.g. *PLN*) [2, 66–70] (Fig. 1). The impact of these variants was elucidated by Escobar-Lopez et al., whose study analysed an extensively characterised cohort of 1,203 genotyped patients with non-ischaemic DCM. Important prognostic signals were observed in this study, where malignant VAs occurred in nearly 30% of patients harbouring high-risk arrhythmic genotypes. In multivariate regression models adjusted for LVEF and NYHA class, both the genotype and LGE were strong independent predictors of malignant VAs [71].

A study by Stroeks et al. utilising unsupervised machine learning on a multicentre cohort of 534 genotype-positive DCM patients further highlights the importance of considering the genetic architecture in arrhythmic risk stratification. By contrasting a “phenotype-first” clustering approach (grouping patients by clinical and imaging similarities) against a “genotype-first” approach, the analysis revealed phenotypic clusters correlated poorly with the underlying genotype [72]. More importantly, the genotype-first approach was vastly superior in predicting clinical outcomes, including malignant VAs. These findings collectively highlight that genetic architecture and CMR-derived tissue characterisation are not independent risk domains but complementary layers of the same arrhythmic risk spectrum: the genotype defines susceptibility, while LGE defines the structural platform through which this susceptibility can produce arrhythmia.

## Limitations

Despite multiple studies highlighting the capabilities of CMR in arrhythmic risk stratification, its widespread implementation faces several limitations. CMR is highly resource-intensive, requiring costly infrastructure, specialised scanning protocols and clinicians trained in reading and interpreting imaging. Indeed, access is often restricted to tertiary academic centres, creating geographical disparities [73]. Additionally, the extensive data linking CMR-defined fibrosis to SCD is predominantly derived from retrospective and prospective observational cohorts. There remains a critical absence of large-scale RCT data showing ICD implantation based primarily on CMR parameters such as the presence of LGE definitively improves overall survival compared to current standard of care.

A major technical hurdle is the lack of consensus regarding the standardisation of fibrosis quantification methodologies. Many methodologies exist for quantifying fibrosis, and while a relative scar burden or total mass cutoff provides compelling prognostic arrhythmic risk stratification in individual studies, there is no universally accepted methodology. These include manual visual scoring methods and various semi-automated signal thresholding techniques such as the full-width at half-maximum (FWHM) method and the *n*-standard deviation (e.g. 2-SD, 5-SD) approaches calculated above normal remote myocardium. This lack of technical standardisation introduces substantial inter-observer, software and inter-vendor variability, making it clinically challenging to uniformly apply absolute quantitative cutoffs across different institutions.

## Future Directions

The evolving landscape of risk stratification in NICM is shifting away from a singular reliance on LVEF and global functional metrics, and towards a more refined approach. This evolution focuses on the direct visualisation of the pro-arrhythmic substrate, tissue-level characterisation on CMR and the integration of multi-dimensional data to better identify patients who will derive a true benefit from ICD therapy.

### Emerging CMR Modalities

Emerging CMR techniques may offer the potential to further refine this substrate-driven approach by looking beyond macroscopic scarring. Advanced microstructural imaging such as diffusion-tensor cardiovascular magnetic resonance (DT-CMR) maps the three-dimensional spatial alignment of myocardial fibres and sheetlets, allowing for cardiac microstructure to be studied at the level of the cardiomyocyte. While existing studies have demonstrated significant microstructural abnormalities in DCM patients, the specific utility of DT-CMR to predict SCD has not yet been formally assessed. Some existing work in HCM has shown early promise for DT-CMR as an emerging imaging biomarker of arrhythmic risk [74].

### Ongoing Clinical Validation

Central to these efforts are several clinical trials designed to move the field towards a more personalised, substrate-driven model of care. The ongoing BRITISH trial (ClinicalTrials.gov Identifier: NCT05568069) is a multicentre, UK-based randomised controlled trial (RCT). This study will enrol 1,252 patients with NICM and LVEF ≤ 35% with LGE on routine CMR. Eligible patients will be randomised to either an interventional arm receiving an ICD or a control arm receiving an implantable loop recorder (ILR) or cardiac resynchronisation therapy pacemaker (CRT-P). The primary endpoint is the comparison of all-cause mortality [75]. Parallel efforts are also being made, where the CMR-GUIDE HF trial (ClinicalTrials.gov Identifier: NCT03993730) is extending this investigational paradigm to patients with mild-to-moderate systolic dysfunction (LVEF 36-50%) with LGE detected on CMR [76].

## Conclusions

The paradigm for arrhythmic risk stratification in NICM is undergoing a fundamental transition from functional assessment to substrate-based precision medicine. CMR is now established as an effective modality in delivering this. Importantly, the convergence of genotype-specific data with CMR imaging holds significant promise by combining structural remodelling and genetic susceptibility as synergistic layers of risk. While the results of landmark trials like BRITISH and CMR-GUIDE HF are still awaited, the current body of evidence suggests that an integrated, multiparametric approach, centred around CMR, is the future of personalised SCD prevention in non-ischaemic cardiomyopathies.

## Data Availability

No datasets were generated or analysed during the current study.

## Key References

- Gulati A, Jabbour A, Ismail TF, Guha K, Khwaja J, Raza S, et al. Association of Fibrosis With Mortality and Sudden Cardiac Death in Patients With Nonischemic Dilated Cardiomyopathy. JAMA. 2013;309:896–908. https://doi.org/10.1001/jama.2013.1363.
  - ○ This was the landmark observational study demonstrating a powerful association between the presence of myocardial fibrosis, identified by late gadolinium enhancement CMR, and sudden cardiac death.
- Hammersley DJ, Zegard A, Androulakis E, Jones RE, Okafor O, Hatipoglu S, et al. Arrhythmic Risk Stratification by Cardiovascular Magnetic Resonance Imaging in Patients With Nonischemic Cardiomyopathy. JACC. American College of Cardiology Foundation; 2024;84:1407–20. https://doi.org/10.1016/j.jacc.2024.06.046.
  - ○ This large 2-centre observational study demonstrated that quantification of scar, identified by LGE-CMR, further enhances arrhythmic risk stratification in NICM. LVEF was found to be an imprecise predictor of arrhythmic risk.
- Eichhorn C, Koeckerling D, Reddy RK, Ardissino M, Rogowski M, Coles B, et al. Risk Stratification in Nonischemic Dilated Cardiomyopathy Using CMR Imaging. JAMA. 2024;332:1535–50. https://doi.org/10.1001/jama.2024.13946.
  - ○ This large meta-analysis confirms the strong association between fibrosis presence and extent with arrhythmic outcomes, across a large number of studies.

## Declarations

### Use of Artificial Intelligence

No artificial intelligence tools were used in the preparation, writing, analysis, or editing of this manuscript.
